# Computational Analysis of the Hypothetical Protein P9303_05031 from Marine Cyanobacterium *Prochlorococcus Marinus* MIT 9303

**DOI:** 10.1515/jib-2018-0087

**Published:** 2020-03-30

**Authors:** PV Parvati Sai Arun, Vineetha Yarlagadda, Govindugari Vijaya Laxmi, Sumithra Salla

**Affiliations:** Department of Biotechnology, Chaitanya Bharathi Institute of Technology, Gandipet, Hyderabad, Telangana 500075, India

**Keywords:** Hsp 10, hypothetical protein, P9303_05031, *Prochlorococcus marinus* MIT 9303, bioinformatics techniques

## Abstract

*Prochlorococcus marinus* MIT 9303 is a marine cyanobacterium found in sea waters. It was first isolated from a depth of 100 m in the Sargasso Sea in the year 1992. This cyanobacterium serves as a good model system for scientific research due to the presence of many desirable characteristics like smaller in size, ability to perform photosynthesis and the ease of culture maintenance. The genome of this cyanobacterium encodes for about 3022 proteins. Out of these 3022 proteins, few proteins were annotated as hypothetical proteins. We performed a computational study to characterize one of the hypothetical proteins “P9303_05031” to deduce its functional role in the cell using various bioinformatics techniques. After in-depth analysis, this hypothetical protein showed the conserved domain as of Hsp10 of molecular chaperonins of GroES. In this work, we have predicted the bidirectional best hits for the hypothetical protein P9303_05031 followed by the prediction of protein properties such as primary, secondary and tertiary structures. The existence of the Hsp10 domain indicates its role is essential for the folding of proteins during heat shock. This work represents the first structural and physicochemical study of the hypothetical protein P9303_05031 in *Prochlorococcus marinus* MIT 9303.

## 1 Introduction

Cyanobacteria are the ancient group of oxygenic photosynthetic micro-organisms existing on earth since 2.7 billion years ago [1]. As they can perform photosynthesis they are considered to be the progenitor of chloroplast present in plants [2]. Cyanobacteria contribute greatly to primary production by fixing a substantial amount of available carbon even in nutrient-limited niches such as oligotrophic marine surfaces to desert crusts [3], [4]. As Cyanobacteria possess vital metabolic pathways and being global producers of carbon and nitrogen budgets, they became one of the widely studied microbes [5]. Cyanobacteria have wide morphological differences from unicellular to filamentous, and also have diverged adaptations such as freshwater, marine water, terrestrial, etc. [6]. Genome sequencing of cyanobacteria was first initiated by sequencing the genome of cyanobacterium *Synechocystis* sp PCC 6803 in the year 1996 [7]. Till today there are several genomes of cyanobacteria sequenced and made publicly available at NCBI (ftp://ftp.ncbi.nlm.nih.gov/genomes). Using these completely sequenced genomes and by applying bioinformatics techniques one can find answers for many questions related to evolution, adaptation, physiology, and biochemistry of cyanobacteria [5]. As this cyanobacterium possesses many hypothetical proteins, characterization of these hypothetical proteins is an important task. For characterization of any protein, there are two approaches followed, namely the experimental approach and computational approach. Experimental approaches are the ones that may have many steps involved, laborious, time taking and costly. There are also many opinions about the experimental studies that sometimes they end up with no results (such as expressing the protein in inclusion bodies, etc.). To counteract these problems, the use of computational methods has gained importance. As there is an enormous amount of data present in publicly available databases, making use of such data would help in the characterization of proteins using computational methods. Generally, for computational characterization of any hypothetical protein, the following steps were performed such as prediction of Physico-chemical proteins, prediction of secondary structure, and prediction tertiary structure [8], [9]. In this report, we have selected a hypothetical protein of a cyanobacterium *Prochlorococcus marinus* MIT 9303.

*Prochlorococcus marinus* MIT 9303 is a marine cyanobacterium. *Prochlorococcus marinus* is abundantly found and dominates the mid-latitude of oceans. It was reported to be the smallest known oxygenic phototroph [10]. Numerous isolates of *Prochlorococcus* strains were isolated from different sea waters around the world and deposited in different culture collection centres. The studies performed on these isolated *Prochlorococcus* show that the strains of *Prochlorococcus* are physiologically and genetically distinct from each other and also exist diverse in these areas [10]. Further, all these isolates were assigned into two clades and named them as the “High light” adapted clade, which exists on the surface of the ocean and the other as the “low-light” adapted clade, which is found in ocean depths. At the time of initiation of this work, there were about 12 *Prochlorococcus* strains were identified. The whole-genome sequence of these 12 genomes was completely sequenced and made available in public databases such as NCBI. The cyanobacterium *Prochlorococcus* has several features such as smaller genome size, autotrophic nature, simple regulatory system, the existence of genomic variants, ease of handling made *Prochlorococcus* as a good model system for scientific research [11].

## 2 Materials and Methods

### 2.1 Selection and Downloading Genome Sequences

**Figure 1:**
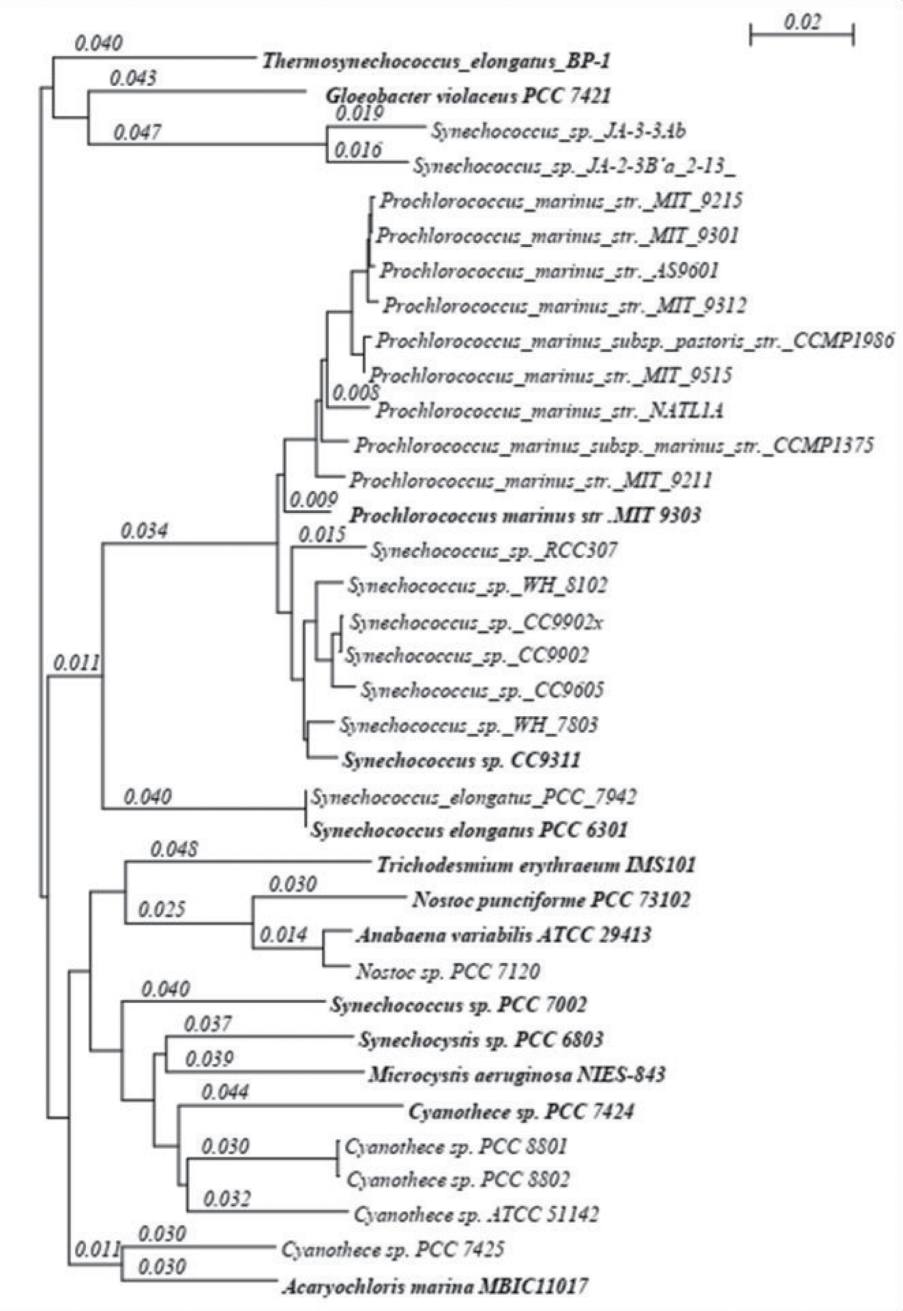
Phylogenetic tree of 16s RNA of 36 bacterial species sequenced at the time of initiation of this work. The bold ones are the species which were selected for our analysis based on the largest genome size.

Based on the 16s RNA phylogenetic tree of Cyanobacteria, Thirteen Cyanobacterial genomes were selected from a total of 36 sequenced cyanobacterial genomes available at the time of initiation of this work (Figure 1). The whole-genome and proteome content of the selected bacteria were downloaded from NCBI. We have considered the cyanobacterial species/strains with the largest genome size among the multiple species/strains of the same genus.

### 2.2 Prediction of Clusters of Orthologous Genes in *Prochlorococcus Marinus* MIT 9303

Clusters of orthologous genes of *Prochlorococcus marinus* MIT 9303 (Hereafter referred as pmmCOGs) were predicted by applying the bidirectional best hit method using BLASTP [12]. Out of many pmmCOG’S generated, we have selected the *pmmCOG P9303_05031* for our analysis.

### 2.3 Prediction of Physico-Chemical Properties for the Proteins of *pmmCOG P9303_05031*

We used the PEPSTATS tool provided in the EMBOSS package (http://emboss.bioinformatics.nl/cgi-bin/emboss/pepstats) [13] for the prediction of Physico-chemical properties of the selected COG. The Physico-chemical properties like molecular weight, number of residues, isoelectric point (pI), molar extinction coefficient and amino acid composition of a protein and others were provided by PEPSTATS. We developed in house Perl programs, which use the mathematical equations published earlier for the calculation of Probability of Expressed Protein entering into Inclusion Bodies (PEPIB), Aliphatic Index, and GRAVY value as described in the database CyanoPhyChe [5]. We have taken the PEPSTATS output as input for calculation of Aliphatic Index and GRAVY, and PEPIB.

### 2.4 Prediction of Secondary Structure

All the protein sequences of *pmmCOG P9303_05031* were subjected to secondary structure prediction using PREDATOR [14]. PREDATOR accepts the input protein sequence in the form of a FASTA formatted file and then predicts the secondary structure using profiles present in the STRIDE database of PREDATOR.

### 2.5 Domain Search and Protein Family Identification

We did Pfam [15] and ProDom [16] searches for the identification of protein families and the conserved domains for assigning a putative function for the proteins in *pmmCOG P9303_05031*.

### 2.6 Developing Tertiary Structure of the Protein

The tertiary structure of the query protein was developed using MODELLER version 13 [17].

### 2.7 Generating Ramachandran Plot

Tertiary structure validation was done by developing the Ramachandran plot using the RAMPAGE server [18]. Visualization of the built 3D structure obtained from homology modelling, superimposition and calculation of RMSD value between the built structure and its template was done in PyMOL [19].

### 2.8 Prediction of Protein-Protein Interaction

The protein sequence of the query protein was downloaded from the CyanoPhyChe database [5] in FASTA format. The downloaded protein sequence was then given as input to the STRING database [20] for the prediction of protein-protein interactions.

## 3 Results and Discussion

The strain of the current study *Prochlorococcus marinus* MIT 9303 was isolated from a depth of 100 m at the Sargasso Sea in 1992. This strain is low-light adapted strain has a total 2,682,807 nucleotides base pairs with 50.1% GC content. It has a total of 3022 genes of coding for different proteins with both known and hypothetical functions [10].

### 3.1 Ortholog Clusters of *pmmcog p9303_05031*

Upon performing homology searches, we derived the first clue about the protein coded by the gene *P9303_05031*. From Table 1, we observed that the function of bidirectional best hits among the other cyanobacteria with respect to the selected hypothetical protein encoded by *P9303_05031* is found to be chaperonin/ co-chaperonin GroES.

**Table 1:** Table representing names of the genomes, their bidirectional best hit and its function among different cyanobacterial genomes.

Name of the genome	Bidirectional best hits	Function
* Prochlorococcus marinus* MIT 9303	* P9303_05031*	Hypothetical protein
* Acaryochloris marina* MBIC 11017	* Am1_4412*	Chaperonin GroES
* Anabaena variabilis* ATCC 29413	* Ava_3627*	Co-chaperonin GroES
* Cyanothece* PCC 7424	* Pcc7424_1789*	Co-chaperonin GroES
* Gloeobacter violaceus* PCC 7421	* Gvip396*	Co-chaperonin GroES
* Microcystis aeruginosa* NIES 843	* Mae_46070*	Co-chaperonin GroES
* Nostoc punctiforme* PCC 73102	* Npun_r0830*	Co-chaperonin GroES
* Synechococcus* CC 9311	* Sync_2283*	Co-chaperonin GroES
* Synechococcus elongatus* PCC 6301	* Syc1788_d*	Co-chaperonin GroES
* Synechococcus* JA 2 3B a 2 13	* Cyb_1619*	Co-chaperonin GroES
* Synechococcus* PCC 7002	* Synpcc7002_a2457*	Co-chaperonin GroES
* Synechocystis* PCC 6803	* Slr2075*	Co-chaperonin GroES
* Thermosynechococcus elongatus* BP1	* Tll0186*	Co-chaperonin GroES
* Trichodesmium erythraeum* IMS 101	* Tery_4326*	Co-chaperonin GroES

The highlighted one is our genome and gene of interest with hypothetical function.

### 3.2 Physico-Chemical Properties of Hypothetical Protein P9303_05031 and its Orthologs

The Physico-chemical properties analysis revealed that the hypothetical protein has a total of 166 amino acids in its sequence. The molecular weight of the protein was found to be 17463.79 daltons. The theoretical iso-electric point was found to be 6.09. The maximum number of amino acids present in the sequence was found to be that of Glycine (G) (10%). The least number of amino acids present in the sequence was Methionine (M) (1.2%). The total number of positively charged residues (Arginine and Lysine) is 16 and the negatively charged residues (Aspartic acid and Glutamic acid) are 19. The GRAVY was calculated to be −0.13. The predicted aliphatic index was found to be 86.2. The significance of an aliphatic index is that the more the value the higher stability towards temperature. The probability of expressed entering into inclusion bodies (PEPIB) was found to be 0.193, which means that, if this gene is cloned into *E. coli* and if subjected for its heterologous expression, then the probability of this protein getting expressed into the soluble fraction (the supernatant) is more than that of the protein entering into inclusion bodies. The other details of the Physico-chemical properties of the hypothetical protein and its orthologs are presented in Supplementary Table 1.

### 3.3 Secondary Structure Elements

**Figure 2:**
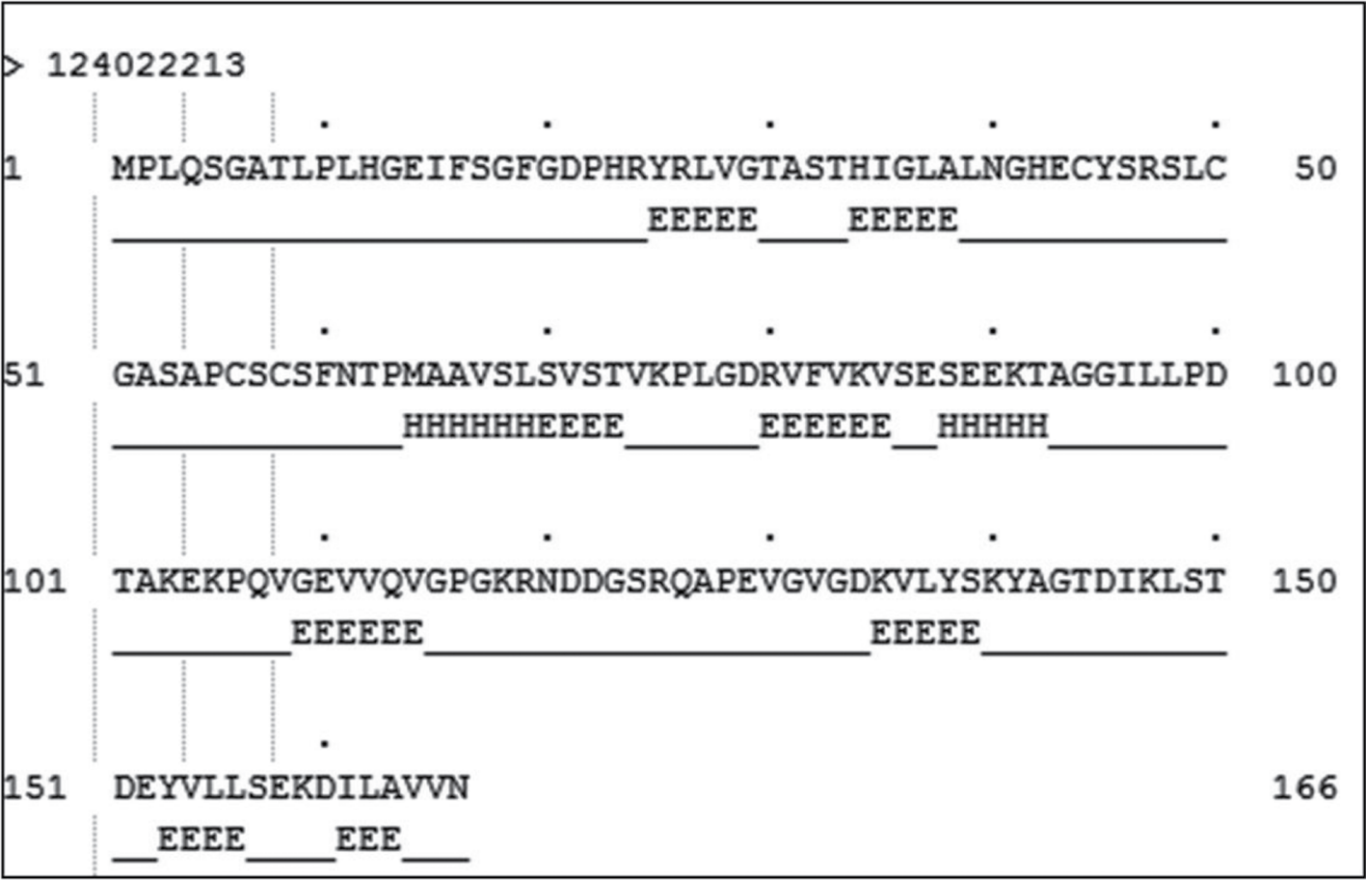
Secondary structure prediction for the protein P9303_05031. Secondary structure was predicted for all 166 amino acids present in the protein sequence. The Helical regions are shown as “H”, the coiled regions are shown as “–––” and the Sheets are shown as “E”.

The secondary structure analysis of the protein was done as described in materials and methods. From the secondary structure analysis (Figure 2), it was observed that the distribution of the total number of amino acids in the coils is about 70.5%, whereas in helices and Sheets there are about 6.7% and 22.8% respectively.

### 3.4 Domain Search and Protein Family Identification

**Figure 3:**
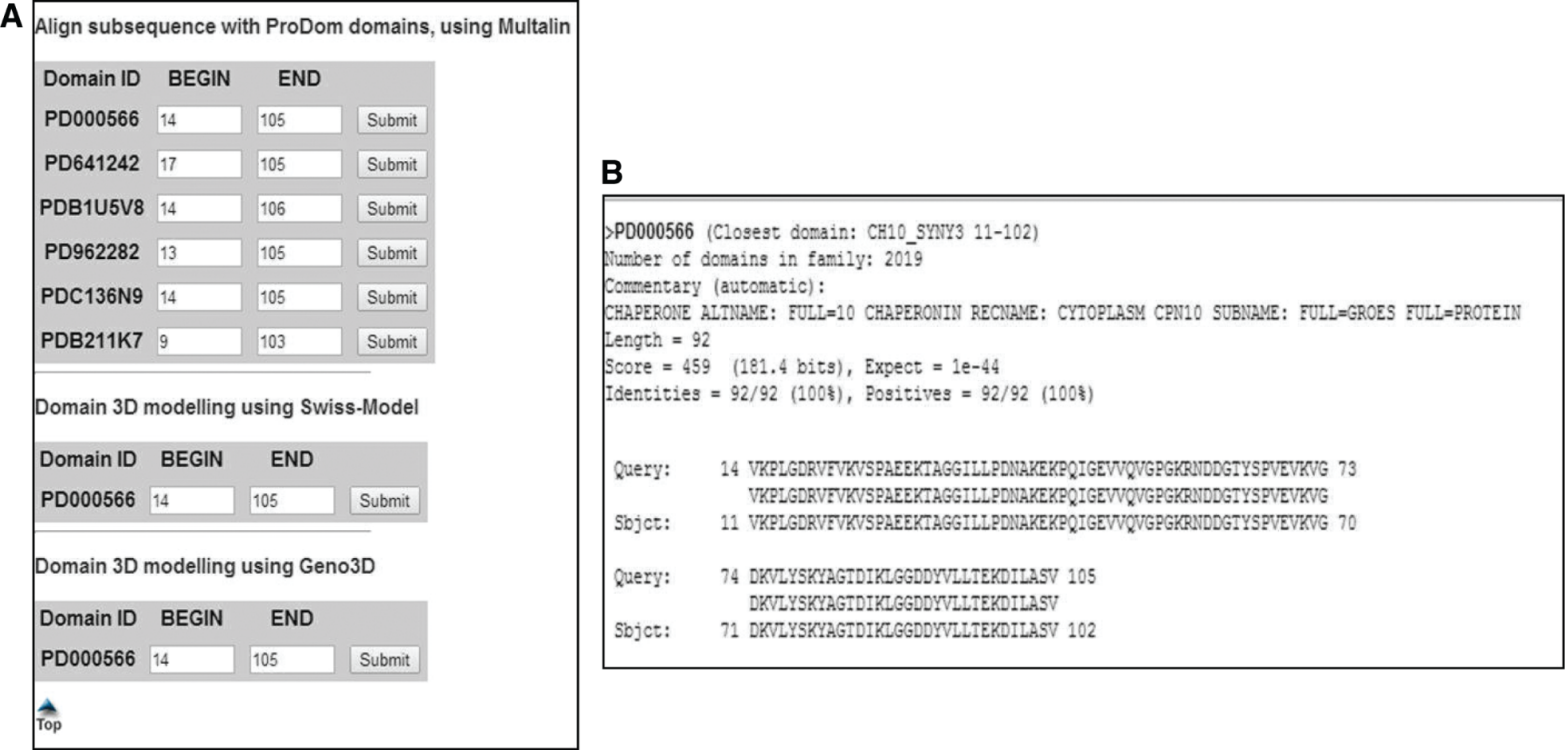
Predicted secondary structure of our protein of interest. (A) The output of ProDom searches against the query protein P9303_05031. It is clear from that PD000566 is the first best hit in the ProDom search. (B) Represents the alignment of the PD000566 with our query protein P9303_05031. From the above alignment it is clear that from residue 14 to 105 the entire amino acids stretch is conserved between PD000566 and the query protein.

Pfam is a database of protein families. Pfam also includes multiple sequence alignments of protein families that are generated using Hidden Markov models. We have selected the link “Sequence search” (second option) available in the Pfam database website for the identification of the conserved domains. From Pfam domain analysis, we observed that the hypothetical protein P9303_05031 has a chaperonin 10kd subunit in its proteins sequence and it belongs to the cnp10 family. We also used the ProDom database for additional analysis composed of protein domains families. ProDom has the capability of constructing homologous segments of protein domains by clustering. The building procedure MKDOM2 of ProDom is based in Position-Specific Iterative BLAST. The entries present in ProDom are in the form of multiple sequence alignments of homologous domains and with a consensus sequence. Figure 3, shows the best matches of the ProDom database with the hypothetical protein in question. Here the best match is found to be PD000566. PD000566 is the ID given to the chaperonin 10kd subunit in the ProDom database. By observing the results obtained from Pfam searches and ProDom searches, it is evident that the hypothetical protein has cpn10 domain conserved in it.

### 3.5 Tertiary Structure of Hypothetical Protein P9303_05031

**Figure 4:**
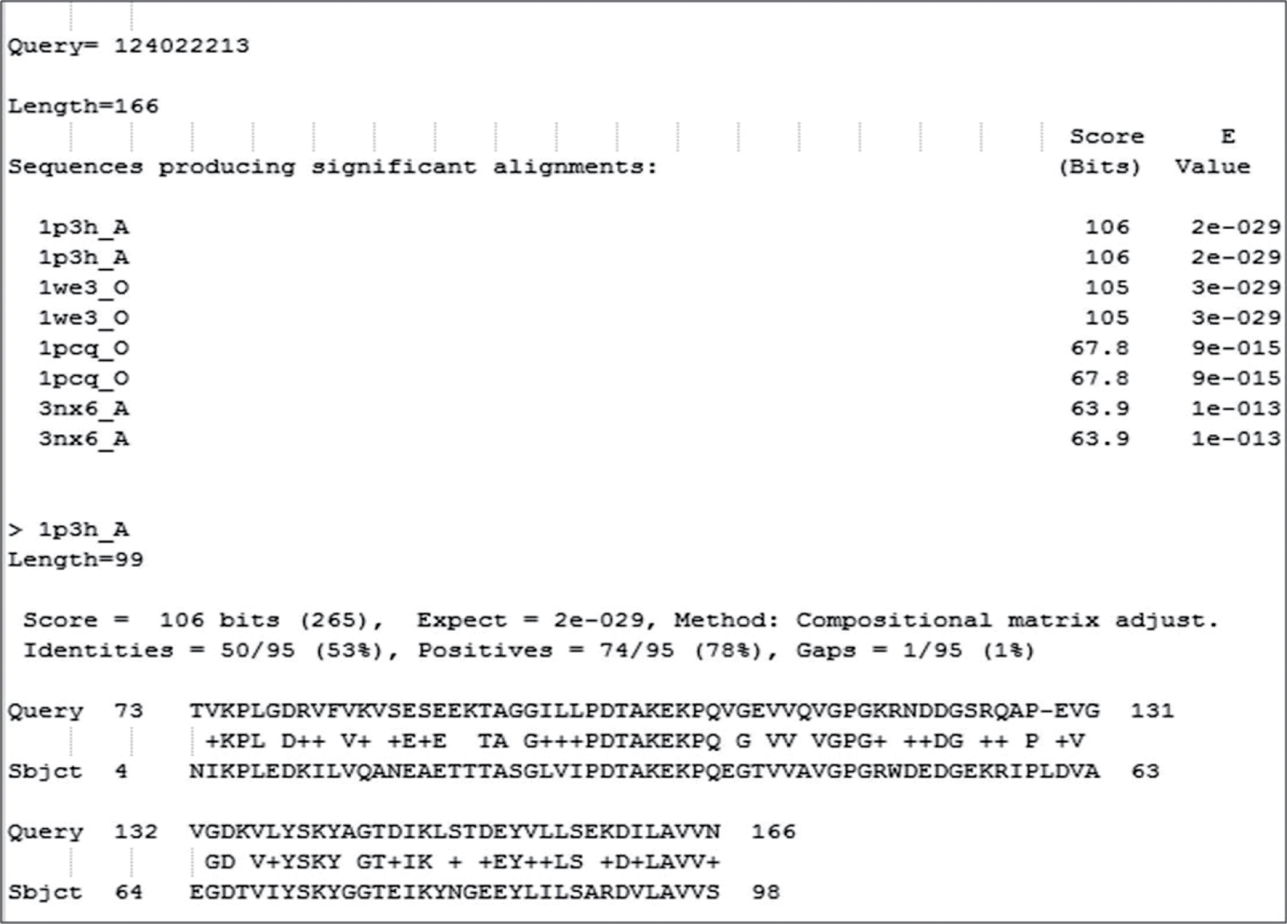
BLASTP search against PDB database and hypothetical protein P9303_05031. The first top hit is found to be 1P3H_A. Below the hits list, the alignment between the protein P9303_05031 and 1P3H chain A is can be found. The percentage identity between 1P3H’s chain A and P9303_05031 protein is found to be 53%.

We build the tertiary structure of the protein in question using homology modelling. As homology modelling technique requires a template, we searched the Protein data bank for the best template. We obtained the PDB “1P3H” as a good template for building the model for the hypothetical protein. The template is from the organism *Mycobacterium tuberculosis.* This 1P3H is the crystal structure of the chaperonin complex. It had 14 chains in it. The protein sequence of P9303_05031 is matching with the chain “A” of 1P3H with a sequence identity of 53% (Figure 4).

**Figure 5:**
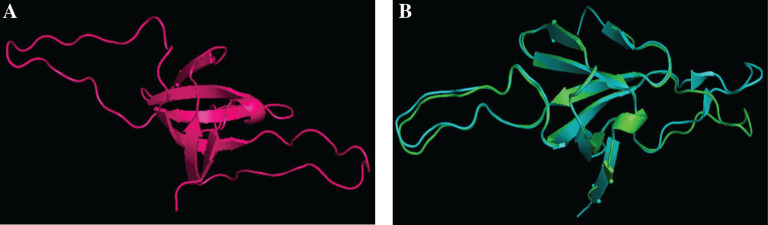
Predicted secondary structure of our protein of interest. (A) Representing the modeled structure of the query protein P9303_0.5031. (B) Show the super imposed structures of the modeled P9303_05031 and its template. Our modelled template is exactly super imposed on the chain A of 1P3H.

For modelling a protein, the general principle is that the percentage identity between the query and the template must not be less than 30%. Here, we have enough percentage identity of 53% to build the model. Further proceeding with the homology modelling, we obtained the structure of P9303_05031 protein (Figure 5A). We superimposed the predicted structure with the chain A of the template and calculated the root mean square deviation. When the predicted structure of the hypothetical protein P9303_05031 super-imposed (Figure 5B), then the RMSD value is found to be 0.387.

### 3.6 Ramachandran Plot Assessment of the Predicted Structure

**Figure 6:**
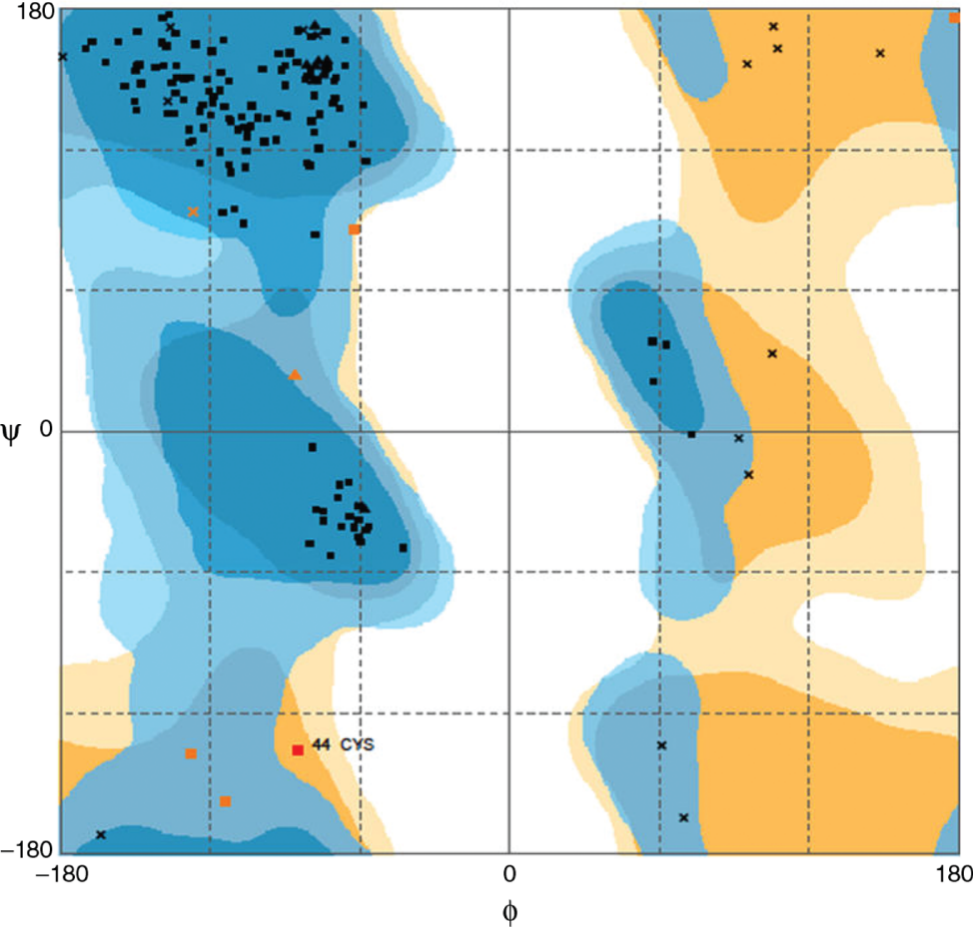
Ramachandran plot analysis for the predicted structure. It was observed that about 157 (95.7%) amino acids are in favourable regions. The total number of residues in the allowed region are 6 (3.7%). The total number of residues outlier region is 1 (0.6%).

As described in material and methods, we used the RAMPAGE server for generating Ramchandran plot for the predicted structure (Figure 6). From Figure 6, it is clear that the total residues in the favoured region are found to be 157 (95.7%). The total numbers of residues in the allowed region are 6 (3.7%). The total number of residues outlier region is 1 (0.6%).

### 3.7 Protein-Protein Interactions

From Protein-Protein interactions, it was found that the hypothetical protein P9303_05031 is in interaction with the proteins such as HrcA, HtpG, GroES, GrpE, DnaJ3, ClpB1, DnaK, DnaK2, GroEL, GroL1, and RpL12 [21], [22], [23], [24], [25]. Upon in-depth literature search, it was found that most of the proteins that interact with the query protein are involved in heat shock response (Table 2). Moreover, the interaction of rpL12 is out of the interactions of the core of Hsps which may be ignored.

**Table 2:** Table showing the functions of the protein which are in interaction with the query protein hypothetical protein P9303_05031. Most of the proteins which are in interaction with the query protein were annotated as the proteins which involve in heat shock response and regulation.

Name of the protein	Function	Reference no
hrcA	Heat shock regulation	[21]
htpG	Heat shock protein	[22]
groES	Heat shock response	[23]
grpE	Heat shock response	[24]
dnaJ3	Heat shock response	[24]
dnak2	Heat shock response	[24]
groEL	Heat shock response	[23]
groL1	Heat shock regulation	[25]
rpl12	Interaction is out of the core of Hsps	–

## 4 Conclusion

The analysis of the hypothetical protein showed sequence similarity mostly to the chaperonin 10kd subunit which belongs to Heat shock proteins family. By comparing the annotations and the sequences of bidirectional hits obtained from BLASTP searches indicates that the protein has the similar function as that of other cyanobacterial GroES proteins. The domain identified from Pfam and ProDom searches in the protein was characteristics of the cnp10 family domain found in a various diverse group of protein which act as Heat shock proteins. The dominance of coiled regions indicates the high level of conservation and stability of the protein structure. Moreover, the protein-protein interactions also show that the protein is to interact with the hub of Hsps which are responsible for adaption of the survival mechanism of bacteria during heat stress. All these above results lead to a conclusion that the hypothetical protein encoded by the gene *P9303_05031* in the marine cyanobacterium *Prochlorococcus marinus* MIT 9303 may encode for GroES kind of protein which is responsible for heat shock response.

**Figure 7:**
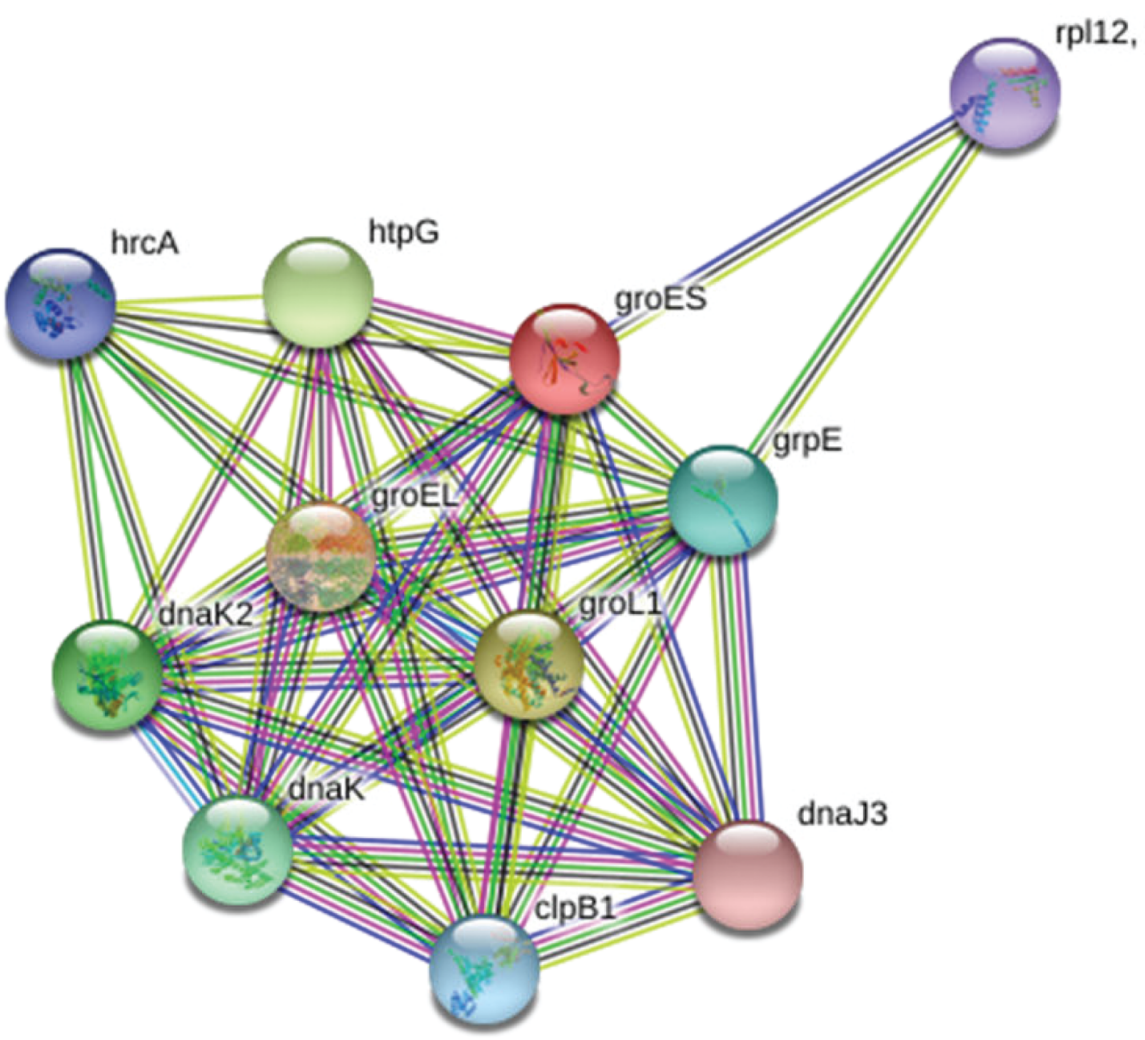
Snapshot showing the STRING database interaction of the query protein with the other proteins of the *Prochlorococcus marinus* MIT 9303. Most of the proteins those interact with the query protein are found to he Hsps involved in heat shock response.
